# Nonlinear Intelligent Control of Two Link Robot Arm by Considering Human Voluntary Components

**DOI:** 10.3390/s22041424

**Published:** 2022-02-12

**Authors:** Mingcong Deng, Shotaro Kubota, Yuanhong Xu

**Affiliations:** Department of Electrical and Electronic Engineering (The Graduate School of Engineering), Tokyo University of Agriculture and Technology, 2-24-16 Nakacho, Tokyo 184-8588, Japan; 1212kubota@gmail.com (S.K.); xuyuanhongdp@gmail.com (Y.X.)

**Keywords:** nonlinear intelligent control, support vector regression, feedforward control, human arm viscoelastic

## Abstract

This paper proposes a nonlinear intelligent control of a two link robot arm by considering human voluntary components. In general, human arm viscoelastic properties are regulated in different manners according to various task requirements. The viscoelasticity consists of joint stiffness and viscosity. The research of the viscoelasticity can improve the development of industrial robots, rehabilitation and sports etc. So far, some results have been shown using filtered human arm viscoelasticity measurements. That is, human motor command is removed. As a result, the dynamics of human voluntary component during movements is omitted. In this paper, based on the feedforward characteristics of human multi joint arm, a model is obtained by considering human voluntary components using a support vector regression technique. By employing the learned model, a nonlinear intelligent control of two link robot arm is proposed. Experimental results confirm the effectiveness of this proposal.

## 1. Introduction

In recent years, in the medical and welfare fields, human resources with appropriate skills are required for treatment/surgical support for patients and long-term care for the elderly. However, the shortage of human resources due to the declining birthrate and aging population has become a problem. As one of the solutions to the above problems, it is conceivable to adopt robots as a labor force. In the future, the places where robots will be active in society will increase not only in factories, but also in facilities and general households where they have contact with humans, so it is necessary to operate robots in cooperation with humans. Therefore, it is desirable that the robot has an excellent man-machine interface, has an affinity with humans, and has the same kinetic characteristics as humans.

Multi-joint viscoelastic properties are attracting attention as an elucidation of human motion control principles. Human arm multi-joint viscoelasticity is the characteristic of the arm joint when the human arm comes into contact with the outside world. The torque that moves a human skeletal joint is generated by the difference in tension between the leading and competing muscle groups. The above tension difference is caused by the activity of muscles controlled by commands from the central nervous system. Muscle control is used not only to generate the joint torque required for exercise, but also to change the stiffness of joints during exercise and at rest. The hardness of the joints mentioned above plays an important role in stabilizing posture and interacting with the outside world [1]. For example, when a person performs a movement to move a cup, the target is mediated by the arm. In addition to interacting with objects, it is also affected by the multi-joint viscoelasticity of this arm. In other words, it is thought that humans perform the desired movement by adjusting the multi-joint viscoelasticity so that the operating environment interacts with the arm and the object. Therefore, learning the work is not only learning the work procedure, but also learning how to control the viscoelastic properties of the above-mentioned musculoskeletal system and contact with the outside world. Exploring the mechanism of adjusting the joint mechanical impedance of the musculoskeletal system is to elucidate the motor control principle of the brain that controls complex multi-joint movements, quantitatively understands the deterioration of movements caused by nerve and muscle disorders, and has human-friendly mechanical interfaces. It can be said that it is an important issue in the development of [2].

Based on the above, many studies on human arm multi-joint viscoelasticity have been conducted [3,4,5,6,7,8,9,10,11]. In 1998, Gomi et al. proposed a method for estimating the viscoelasticity of the human arm during exercise using a Kalman filter [12]. Furthermore, Deng et al. focused on the numerical instability caused by the Kalman filter’s digit loss, and he proposed the adaptation of the UD decomposition method as a solution. Based on these previous studies, Wang proposed motion control of a robot arm considering human arm multi-joint viscoelasticity, and its effectiveness was confirmed by simulation [13]. On the other hand, there is no example of applying human arm multi-joint viscoelasticity to the control of an actual robot arm. The problem in applying it to robot arm motion control is the reproduction of voluntary motion components. The voluntary movement component is a feedforward (FF) component output from a model based on experience in the brain when exercising. In the research to actually estimate the multi-joint viscoelasticity of the human arm, the estimation is performed after removing the above voluntary movement components with a filter. Therefore, in order to apply the multi-joint viscoelasticity of the human arm to the robot arm, it is necessary to reproduce the voluntary movement component with a feedforward controller.

In this study, we focus on the reproduction of the above voluntary movement components. Specifically, we aim to design a feedforward controller that has high control performance in the control of the robot arm. Since the feedforward controller proposed in the previous research is designed based on a mechanical model, there is a concern that the control performance will deteriorate due to modeling errors when conducting actual machine experiments. Therefore, in this research, we use Support Vector Regression (SVR) [14,15,16,17,18], which is a kind of machine learning methods, to design the feedforward controller to reduce the modeling error and improve the tracking performance. The control system is designed based on operator theory [19,20,21,22,23,24] to compensate for the interference and uncertainty inside the controlled object that exist when controlling the robot arm. Finally, in order to confirm the effectiveness of the proposed control system, we conduct an actual machine experiment and verify its effectiveness.

In summary, the contributions of this paper are as follows: the viscoelastic properties of the multi-joint arm are measured and analyzed through experiments. Based on the characteristic of multi-joint arm viscoelastic, a controller to simulate the human body is designed, and support vector regression is used for feedforward control.

In what follows, in Section 2, as a mathematical preparation to avoid complicating this paper, Lagrange’s equation of motion used for modeling and SVR theorems used in the design of feedforward control are explained. In Section 3, as a preparation for setting the problem, we introduce the human arm multi-joint viscoelasticity, robot arm modeling and the configuration of the experimental equipment used, and then raise the problem. Section 4 explains the proposed control system, where we explain the design method for the feedback controller using multi-joint viscoelasticity of the human arm, the feedforward controller based on SVR, and the stabilization controller based on the operator theory. Section 5 first describes the experimental conditions and the SVR parameter determination method based on actual machine experiments. After that, experiment is conducted to confirm the effectiveness of the feedforward controller based on SVR. In the absence of a feedforward controller, the experimental results of using a feedforward controller based on a mechanical model and the experimental results of a feedforward controller based on SVR are compared. Section 6 describes the conclusions of this study.

## 2. Mathematical Preparation

In this section, Lagrange’s motion equation, which is necessary when deriving a mechanical model of a robot arm, is explained.

### 2.1. Lagrange’s Equation of Motion

When deriving the equation of motion of an object, Newton’s equation of motion is generally used, but in the case of a complicated mechanical system, it is often difficult to derive it by Newton’s equation. Lagrange’s Equations are often used in the analysis of mechanical systems because they can solve the equations of motion of complex mechanical systems more efficiently than Newton’s equations of motion. However, note that the derived solution does not change from Newton’s equation because it is essentially based on the same physical law as Newton’s equation of motion. In this section, we derive Lagrange’s equation of motion. It is divided into three sections.

#### 2.1.1. Generalized Coordinates and Nonholonomic Constraints

In order to show the dynamic behavior of mass system and rigid system, it is necessary to select physical variables appropriately. In this section, we discuss the mass point system for simplicity, but the same idea is possible for systems including rigid systems. Generally, positions are expressed using plaque points in orthogonal coordinate systems, cylindrical coordinate systems and spherical coordinate systems, but here we consider coordinates that are convenient for expressing the position (arrangement) of the entire plaque point system and defined it as generalized coordinates. A set of generalized coordinates may include parts of a Cartesian or spherical coordinate system, but may also use angles, lengths, distances, and so on.

Now, considering any geometrical arrangement that a given mass system can take, a generalized coordinate system is said to be perfect when any of these arrangements can be represented by giving coordinates. Also, the set of generalized coordinates corresponds to continuous fluctuations in some of the coordinates, whether one of them is removed and all the rest are fixed, or all but some of them are fixed. It is said to be independent when a continuous change in its geometrical arrangement can remain. Taking some generalized coordinates for a very wide class of mass and rigid systems, including robot manipulators such as akrobot, which is the subject of this study. The number of independent coordinates in it is often constant despite changes in the permissible arrangement, which is then called the degree of freedom of the system.

A mass system has less degrees of freedom when it receives a geometric constraint. If the geometric constraint can be expressed analytically by generalized coordinates and an equation that depends only on time, the constraint is nonholonomic. Now, suppose choosing $\left(x_{1},x_{2},\ldots ,x_{m}\right)$ as the complete generalized coordinate system for a mass system, the coordinate system is not independent, there are *p* holonomic constraints such as:

(1) $$\left\{\begin{array}{c} h_{1}\left(x_{1},x_{2},\ldots ,x_{m},t\right)=0\\ h_{2}\left(x_{1},x_{2},\ldots ,x_{m},t\right)=0\\ \ldots \ldots \ldots \ldots \\ h_{p}\left(x_{1},x_{2},\ldots ,x_{m},t\right)=0 \end{array}\right..$$

When these constraints are independent, there are n=m−p independent coordinates out of *m* coordinates. The mass system has *n* degrees of freedom. Therefore, suppose that a generalized coordinate system $\left(q_{1},q_{2},\ldots ,q_{n}\right)$ that is completely and independent from the beginning is selected for the mass system of *n* degrees of freedom. In addition, if part of a complete generalized coordinate system $\left(x_{1},x_{2},\ldots ,x_{m}\right)$ is $\left(q_{1},q_{2},\ldots ,q_{n}\right)$, then the remaining *p* of the former are determined by Equation (1). Assuming that the mass system consists of *N* mass points, it is expressed that the position vector $r_{i}$ of any mass point $m_{i}$ is determined by the generalized coordinate system $\left(q_{1},q_{2},\ldots ,q_{n}\right)$.

(2) $$r_{i}=r_{i}\left(q_{1},q_{2},\ldots ,q_{n},t\right)=r_{i}\left(q,t\right)$$

The velocity $v_{i}$ of this mass point is

(3) $$v_{i}=\frac{d}{dt}r_{i}=\sum_{j=1}^{n}\frac{\partial r_{i}}{\partial q_{j}}{\dot{q}}_{j}+\frac{\partial r_{i}}{\partial t}.$$

This time derivative $\dot{q}=\left({\dot{q}}_{1},{\dot{q}}_{2},\ldots ,{\dot{q}}_{n}\right)$ is called general acceleration.

Since the generalized position coordinate system $\left(q_{1},q_{2},\ldots ,q_{n}\right)$ is complete and independent, the set of infinitesimal variations of coordinates $\left(\delta q_{1},\delta q_{2},\ldots ,\delta q_{n}\right)$ is also complete and independent. Therefore, the variation of the position $r_{i}$ of the quality point $m_{i}$ is represented by the variation $\delta q_{1}$ of the generalized coordinates.

(4) $$\delta r_{i}=\sum_{j=1}^{n}\frac{\partial r_{i}}{\partial q_{j}}\delta q_{j}$$

Next, assuming that the force $f_{i}$ is acting on each mass point $m_{i}$, the increment of all the work done by $f_{i}$ is calculated under the variational $\delta r_{i}$ of the arrangement of the mass system.

(5) $$\sum_{i=1}^{N}f_{i}^{T}\delta r_{i}=\sum_{i=1}^{N}\sum_{j=1}^{n}f_{i}^{T}\frac{\partial r_{i}}{\partial q_{j}}\delta q_{j}=\sum_{i=1}^{N}\left(\sum_{j=1}^{n}f_{i}^{T}\frac{\partial r_{i}}{\partial q_{j}}\right)\delta q_{j}$$

The *j*th on the right side represents the force component in that direction obtained from the infinitesimal variation $\delta q_{i}$ of one of the generalized coordinates $q_{j}$, and this force is called the generalized force.

(6) $$F_{j}=\sum_{j=1}^{N}f_{i}^{T}\frac{\partial r_{i}}{\partial q_{j}}$$

Using the generalization force, (5) is expressed as,

(7) $$\sum_{i=1}^{N}f_{i}^{T}\delta r_{i}=\sum_{j=1}^{n}F_{j}\delta q_{j}.$$

#### 2.1.2. Hamilton’s Principle

If the momentum vector of the mass point $m_{i}$ is set as $p_{i}$, the equation of motion of the mass point system is expressed as

(8) $$f_{i}-\frac{d}{dt}p_{i}=0$$

Note that with the nonholonomic constraint, this equation is redundant and can be expressed for any variation $\delta r_{i}$.

(9) $$\sum_{i=1}^{N}{\left(f_{i}-\frac{d}{dt}p_{i}\right)}^{T}\delta r_{i}=0.$$

However, since $\delta r_{i}$ is generally not independent, (8) is (9). Therefore, we derive Hamilton’s principle from (9) and derive *n* independent equations of motion equal to *n* degrees of freedom.

(10) $$\sum_{i=1}^{N}f_{i}^{T}\delta r_{i}$$

In general, the equation represents the sum of the work done by the forces acting on all mass points in the mass system, but it is divided into a part due to conservative force and a part due to non-conservative external force. That is, the potential energy corresponding to the conservative force is V(q), the generalized force is $F_{j}$, and (10) is expressed as the following equation.

(11) $$\sum_{i=1}^{N}f_{i}^{T}\delta r_{i}=-\delta V+\sum_{j=1}^{n}F_{i}\delta q_{j}$$

The first term on the right side is the decrease in potential energy, and the second term is the work done by the external force. Substituting (11) into (9) gives the following equation.

(12) $$-\delta V+\sum_{j=1}^{n}F_{i}\delta q_{j}-\sum_{i=1}^{N}f_{i}-\frac{dp_{i}^{T}}{dt}\delta r_{i}=0$$

Here, the third term on the left side can be rewritten as

(13) $$-\frac{dp_{i}^{T}}{dt}\delta r_{i}=-\sum_{i=1}^{N}\frac{d}{dt}\left(p_{i}^{T}\delta r_{i}\right)+\sum_{i=1}^{N}p_{i}^{T}\frac{d}{dt}\delta r_{i}.$$

Also, assuming that the mass fluctuation of each mass point of the target mass point system does not occur in the time interval considered, the variation of the total kinetic energy is

(14) $$\delta K=\sum_{i=1}^{N}p_{i}^{T}\frac{d}{dt}\delta r_{i}.$$

Substituting (14) into the right side of (14) palce yields

(15) $$-\frac{dp_{i}^{T}}{dt}\delta r_{i}=-\sum_{i=1}^{N}\frac{d}{dt}\left(p_{i}^{T}\delta r_{i}\right)+\delta K.$$

Substituting (15) into (12) yields,

(16) $$\delta K-\delta V+\sum_{j=1}^{n}F_{i}\delta q_{j}-\sum_{i=1}^{N}\frac{d}{dt}\left(p_{i}^{T}\delta r_{i}\right)=0.$$

This equation holds for any time interval $\left[t_{1},t_{2}\right]$ we are thinking of, so that the variation of position $\delta r_{i}\left(t_{1}\right)=0$ and $\delta r_{i}\left(t_{2}\right)=0$. This is possible because the generalized coordinate system is perfect, and when (16) is integrated over the interval $\left[t_{1},t_{2}\right]$, the fourth term on the right side disappears and the following equation holds.

(17) $$\int_{t_{1}}^{t_{2}}\left(\delta \left(K-V\right)+\sum_{j=1}^{n}F_{i}\delta q_{j}\right)dt=0$$

Equation (17) is called Hamilton’s principle for a nonholonomic mass system with *n* degrees of freedom.

#### 2.1.3. Lagrange’s Equation of Motion

In order to derive an independent equation of motion equal to *n* degrees of freedom from Hamilton’s theorem, we introduce a physical quantity called Lagrangian as in the following equation.

(18) L=K−V

where *K* is the kinetic energy and *V* is the potential energy. Since *V* is the potential energy, it is a function of only the generalized coordinate ${\dot{q}}_{j}$, but *K* is a function of ${\dot{q}}_{j}$, $q_{j}$ and time *t*. Lagradian *L* can be written as,

(19) $$L=L(\dot{q},q,t).$$

The variation is

(20) $$\delta L=\sum_{j=1}^{n}\left(\frac{\partial L}{\partial {\dot{q}}_{j}}\delta {\dot{q}}_{j}+\frac{\partial L}{\partial q_{j}}\delta q_{j}\right).$$

Substituting this into (17) yields,

(21) $$\int_{t_{1}}^{t_{2}}\sum_{j=1}^{n}\left(\frac{\partial L}{\partial {\dot{q}}_{j}}\left(\frac{d}{dt}\right)\delta {\dot{q}}_{j}+\frac{\partial L}{\partial q_{j}}\delta q_{j}+F_{i}\delta q_{j}\right)dt=0.$$

Here, if the first term on the left side is integrated by parts, it can be seen that (21) becomes,

(22) $$\int_{t_{1}}^{t_{2}}\sum_{j=1}^{n}\left(-\frac{d}{dt}\left(\frac{\partial L}{\partial {\dot{q}}_{j}}\right)+\frac{\partial L}{\partial q_{j}}+F_{i}\right)\delta q_{j}dt=0.$$

Since (22) must hold for any variation $\delta q_{j}$, the following *n* equations must hold for the time interval $t\in [t_{1},t_{2}]$. This is the equation of motion of the mass system described in generalized coordinates $q=(q_{1},\ldots ,q_{n})$, and is called the equation of motion of Lagrange.

(23) $$\frac{d}{dt}\left(\frac{\partial L}{\partial {\dot{q}}_{j}}\right)-\frac{\partial L}{\partial q_{j}}=F_{j}$$

Many of the equations of motion of mass and rigid systems with nonholonomic constraints can be derived by using Lagrange’s equation of motion with the following steps.

- Select a complete and independent generalized coordinate system.
- Identify non-conservative generalization forces.
- Find the kinetic energy and potential energy to construct the Lagrangian.
- Substitute Lagrangian into Lagrange’s equation of motion and write down the equation of motion concretely.

### 2.2. Support Vector Regression

Support vector regression is an application of a support vector machine to a regression problem [14,15,16,17,18]. Support vector regression is called SVR and support vector machine is called SVM. SVM is a typical method of binary classification and has a high prediction for unknown data. It has been reported that it is possible to construct a classifier (function) with measurement accuracy. SVM uses methods such as margin maximization and kernel tricks for identification hyperplane design, and SVR is an adaptation of these methods to regression problems. Therefore, SVR has features such as high generalization performance and effectiveness even for those with non-linear input/output relationships. This section describes the procedure for deriving the regression function and the kernel function used for the regression function.

#### 2.2.1. Derivation of Regression Function

This section describes the procedure for deriving the regression function based on SVR. The regression function of SVR is expressed by the following equation.

(24) $$f\left(x\right)=\omega^{T}\phi \left(x\right)+b$$

Let f(x) be the regression function, x be the input vector, ω be the regression coefficient of the feature space, ϕ be the feature function of SVR, and *b* be the bias term. The regression function is determined from the training data using an SVM-based method. In order to determine the regression function, it is necessary to derive the regression coefficient ϕ and the bias term *b*. Let $\left(x_{i},y_{i}\right)$ be the input/output training data used to determine the function of (24). Here, the slack variable is introduced as follows. ϵ is a setting parameter.

(25) $$\begin{array}{cc} \xi_{i}^{+} & =\left\{\begin{array}{cc} 0 & \left(y_{i}-f\left(x_{i}\right)\leq \epsilon \right)\\ y_{i}-f\left(x_{i}\right)-\epsilon & \left(y_{i}-f\left(x_{i}\right)>\epsilon \right) \end{array}\right.\\ \xi_{i}^{-} & =\left\{\begin{array}{cc} 0 & \left(y_{i}-f\left(x_{i}\right)\geq -\epsilon \right)\\ -\epsilon -y_{i}+f\left(x_{i}\right) & \left(y_{i}-f\left(x_{i}\right)<-\epsilon \right) \end{array}\right. \end{array}$$

By using the slack variables $\xi_{i}^{+}$ and $\xi_{i}^{-}$, SVR is formulated as follows.

(26) $$\min_{\omega ,b,\xi }\left[\frac{1}{2}\omega^{T}\omega +C\sum_{i=1}^{n}\left(\xi_{i}^{+}+\xi_{i}^{-}\right)\right]$$

It is assumed that the constraint condition

(27) $$\left\{\begin{array}{ccc} y_{i}-\omega^{T}\phi \left(x_{i}\right)-b & \leq \epsilon +\xi_{i}^{+}, & i=1,\ldots ,n\\ \omega^{T}\phi \left(x_{i}\right)+b-y_{i} & \leq \epsilon +\xi_{i}^{-}, & i=1,\ldots ,n\\ \xi_{i}^{+},\xi_{i}^{-} & \geq 0, & i=1,\ldots ,n \end{array}\right.$$

is satisfied. Here, *C* is the setting parameter and *n* is the number of training data. (26) maximizes the following objective function by introducing the Lagrange multiplier $\lambda_{i}^{+},\lambda_{i}^{-},$$\mu_{i}^{+},\mu_{i}^{-}$.

(28) $$\begin{array}{cc} L_{p} & =\frac{1}{2}\omega^{T}\omega +C\sum_{i=1}^{n}\left(\xi_{i}^{+}+\xi_{i}^{-}\right)\\ \; & -\sum_{i=1}^{n}\left(\mu_{i}^{+}\xi_{i}^{+}+\mu_{i}^{-}\xi_{i}^{-}\right)\\ \; & -\sum_{i=1}^{n}\lambda_{i}^{+}\left(\epsilon +\xi_{i}^{+}-y_{i}+\omega^{T}\phi \left(x_{i}\right)+b\right)\\ \; & -\sum_{i=1}^{n}\lambda_{i}^{-}\left(\epsilon +\xi_{i}^{-}+y_{i}-\omega^{T}\phi \left(x_{i}\right)-b\right) \end{array}$$

Since the optimal solution is the point where the partial derivative of $L_{p}$ with respect to ω, *b*, $\xi_{i}^{+}$, and $\xi_{i}^{-}$ becomes 0, the following equation holds for the optimal solution.

(29) $$\begin{array}{c} \frac{\partial L_{p}}{\partial \omega }=\omega -\sum_{i=1}^{n}\left(\lambda_{i}^{+}-\lambda_{i}^{-}\right)\phi \left(x_{i}\right)=0 \end{array}$$

(30) $$\begin{array}{cc} \frac{\partial L_{p}}{\partial b} & =\sum_{i=1}^{n}\left(\lambda_{i}^{-}-\lambda_{i}^{+}\right)=0 \end{array}$$

(31) $$\begin{array}{cc} \frac{\partial L_{p}}{\partial \xi_{i}^{+}} & =C-\lambda_{i}^{+}-\mu_{i}^{+}=0 \end{array}$$

(32) $$\begin{array}{cc} \frac{\partial L_{p}}{\partial \xi_{i}^{-}} & =C-\lambda_{i}^{-}-\mu_{i}^{-}=0 \end{array}$$

from (29), ω is

(33) $$\omega =\sum_{i=1}^{n}\left(\lambda_{i}^{+}-\lambda_{i}^{-}\right)\phi \left(x_{i}\right)$$

Therefore, (24) is rewritten as

(34) $$f\left(x\right)=\sum_{i=1}^{n}\left(\lambda_{i}^{+}-\lambda_{i}^{-}\right)\phi {\left(x_{i}\right)}^{T}\phi \left(x\right)+b$$

Substituting (29), (31) and (32) into (28) results in the following dual problem.

(35) $$\begin{array}{c} \max_{\lambda_{i}^{-},\lambda_{i}^{+}}L_{p}=\max_{\lambda_{i}^{-},\lambda_{i}^{+}}\left[-\frac{1}{2}\sum_{i=1}^{n}\sum_{j=1}^{n}\left(\lambda_{i}^{+}-\lambda_{i}^{-}\right)\left(\lambda_{j}^{+}-\lambda_{j}^{-}\right)\right.\phi^{T}\left(x_{i}\right)\phi \left(x_{j}\right)\\ +\sum_{i=1}^{n}y_{i}\left(\lambda_{i}^{+}-\lambda_{i}^{-}\right)\left.-\sum_{i=1}^{n}\epsilon \left(\lambda_{i}^{+}+\lambda_{i}^{-}\right)\right] \end{array}$$

$\lambda_{i}^{+}$ and $\lambda_{i}^{-}$ are

(36) $$\sum_{i=1}^{n}\left(\lambda_{i}^{+}-\lambda_{i}^{-}\right)=0,\;0\leq \lambda_{i}^{+},\;\lambda_{i}^{-}\leq C$$

Using the kernel function,

(37) $$K\left(x_{i},x_{j}\right)=\phi {\left(x_{i}\right)}^{T}\phi \left(x_{j}\right)$$

(35) becomes

(38) $$\begin{array}{c} \max_{\lambda_{i}^{-},\lambda_{i}^{+}}\left[-\frac{1}{2}\sum_{i=1}^{n}\sum_{j=1}^{n}\left(\lambda_{i}^{+}-\lambda_{i}^{-}\right)\left(\lambda_{j}^{+}-\lambda_{j}^{-}\right)K\left(x_{i},x_{j}\right)\right.\\ \left.+\sum_{i=1}^{n}y_{i}\left(\lambda_{i}^{+}-\lambda_{i}^{-}\right)-\sum_{i=1}^{n}\epsilon \left(\lambda_{i}^{+}+\lambda_{i}^{-}\right)\right] \end{array}$$

The regression function is obtained from (39) using the kernel function.

(39) $$f\left(x\right)=\sum_{i=1}^{n}\left(\lambda_{i}^{-}-\lambda_{i}^{+}\right)K\left(x_{i},x\right)+b$$

#### 2.2.2. Kernel Function

This section introduces the kernel function used for the regression function. As mentioned above, by using the kernel function, a complicated model can be realized without explicitly calculating ϕ(x). However, not all functions can be used as kernel functions, and it is generally necessary to satisfy Mercer’s theorem. The necessary and sufficient condition for a continuous object and square-integrable function $K\left(x,x^{\prime }\right)$ to have the following expansion for the eigen $\lambda_{i}\geq 0$ and the eigenfunction $\phi_{i}$ is an arbitrary square-integrable function.

(40) $$K\left(x,x^{\prime }\right)=\sum_{i=1}^{\infty }\lambda_{i}\phi_{i}{\left(x\right)}^{T}\phi_{i}\left(x^{\prime }\right)$$

The following conditions are satisfied for *g*.

(41) $$\int_{\chi \times \chi }K\left(x,x^{\prime }\right)g\left(x\right)g\left(x^{\prime }\right)dxdx^{\prime }\geq 0$$

Any function that satisfies the above theorem can be used as a kernel function. In addition, there are many kernel functions that satisfy Mercer’s theorem, and the model learned by changing the kernel function is completely different. Various kernel functions have been proposed according to the application, but this section introduces the basic kernel functions that are used very frequently. There are three basic kernel functions:

(42) $$\begin{array}{cc} K\left(x_{i},x\right) & =x_{i}^{T}x \end{array}$$

(43) $$\begin{array}{cc} K\left(x_{i},x\right) & ={\left(x_{i}^{T}x\right)}^{d} \end{array}$$

(44) $$\begin{array}{cc} K\left(x_{i},x\right) & =\exp\left(-\frac{{∥x_{i}-x∥}^{2}}{2\sigma^{2}}\right) \end{array}$$

The above equations represent a linear kernel, a polynomial kernel and an RBF kernel, respectively. The parameter σ in the RBF function in (44) is expressed as $y_{i}$ as the output data, *N* as the total number of data, and $\bar{y}$ as the average value of the output data.

(45) $$\sigma^{2}=\frac{1}{N}\sum_{i=1}^{N}{\left(y_{i}-\bar{y}\right)}^{2}$$

A linear kernel is a simple kernel function derived when $\phi \left(x_{i}\right)=x_{i}$, but it is often used when a simple model is desired. Both the polynomial kernel and the RBF kernel are capable of implementing non-linear models. Since the above two kernel functions can further adjust the complexity of the model by parameters, in many cases, they are adaptively determined for the data by using cross-validation methods or the like.

## 3. Problem Setup

In this section, the problem setup will be described after explaining the human arm multi-joint viscoelasticity required and the robot arm used in this study.

### 3.1. Human Arm Multi-Joint Viscoelasticity

Human arm multi-joint viscoelasticity is a characteristic that determines the “hardness” of a person’s arm joint. The torque that moves a human skeletal joint is generated by the difference in tension between the leading and competing muscle groups. The above tension difference is caused by the activity of muscles controlled by commands from the central nervous system. On the other hand, muscle control is used not only to generate the joint torque required for exercise, but also to change the “hardness” of joints during exercise and at rest. When both muscle groups between the joints have high tension, the human arm joint becomes “hard”. However, when the tensions of both muscle groups are small, the human arm joint becomes easy to move. The above-mentioned “hardness” of joints has an important role in interaction with the outside world in work and stabilization in posture maintenance.

The behavior of the human musculoskeletal system is often modeled as a spring-damper mass system, including the inherent characteristics of individual muscles and the characteristics of the reflex system. Since the human musculoskeletal system actually has complicated characteristics, the expression method is not unified, but in general, the coefficient of the spring characteristic is the elasticity (stiffness) and damper characteristic of the musculoskeletal system. The coefficient is called viscosity. The coefficient of change in force with respect to change in acceleration is almost determined by the inertia of the musculoskeletal system, so it is called inertia (mass). These three coefficients are collectively called the mechanical impedance parameter of the musculoskeletal system. Among the mechanical impedance parameters, the stiffness is mainly caused by the elastic properties of the muscle, which changes according to the activity level of the muscle. In the “equilibrium position control hypothesis” [25,26], there are models of the musculoskeletal motor system, utilizing the servo system composed of its elastic characteristics and reflection. It is thought that motion and external force are generated by giving the equilibrium position as a motor command. It can also be said that “learning work” is not only learning the work procedure, but also learning how to control the viscoelastic properties of the above-mentioned musculoskeletal system and come into contact with the outside world. Therefore, exploring the adjustment mechanism of the joint mechanical impedance of the musculoskeletal system is to elucidate the movement control principle of the brain that controls complicated multi-joint movements. It can be said that it is an important issue for quantitative understanding of deterioration of movement caused by nerve and muscle disorders and development of human-friendly mechanical interface.

### 3.2. Robot Arm

In this section, we will introduce the robot arm, which is the experimental device handled in this study, and then explain the derivation of the dynamic model. Finally, we will introduce the hardware configuration of the experimental equipment.

#### 3.2.1. Experimental Device

**Figure 1:**
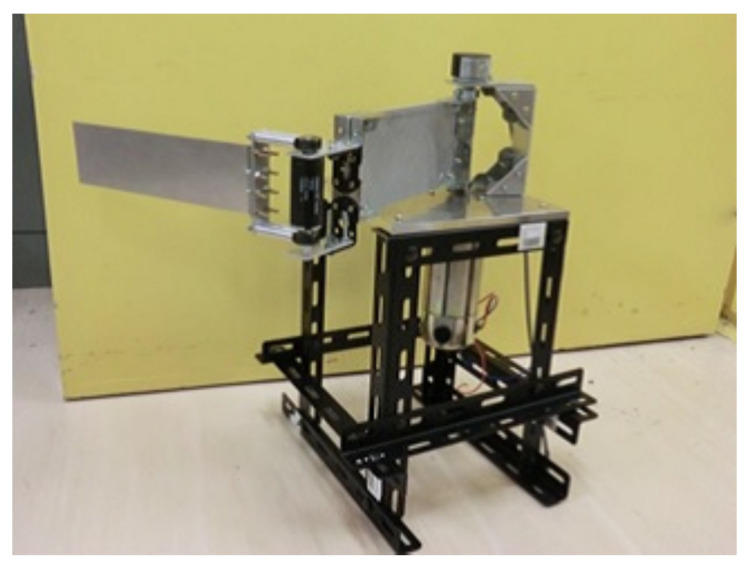
Robot arm used in this research (Mechanical part).

In this research, we conduct an experiment using the two-degree-of-freedom horizontal robot arm that imitates a human arm. Figure 1 shows the horizontal multi-joint robot. It is characterized by using lightweight aluminum for each link and driving each link by a direct drive method.

**Figure 2:**
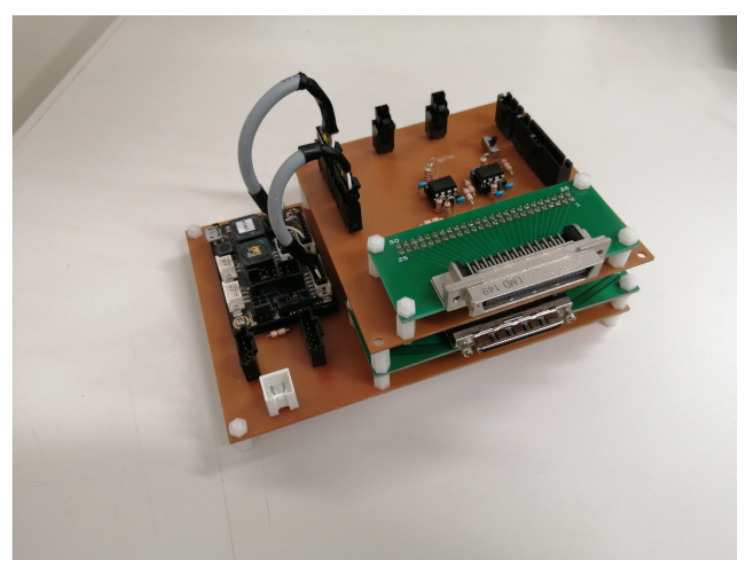
Robot arm used in this research (Electric part).

The self-made buffer circuit used in the experimental equipment of the robot arm is shown in Figure 2. This circuit consists of two boards, the first stage has an input connector for a rotary encoder and an output connector for connecting to a PCI board. The motor controller for the Link 2 motor is also screwed to his first stage, but the power system is separate. In the second stage, the width of the input/output voltage differs between the PCI board and the motor controller, so an amplifier circuit composed of operational amplifiers is built.

#### 3.2.2. Mechanics Modeling

**Figure 3:**
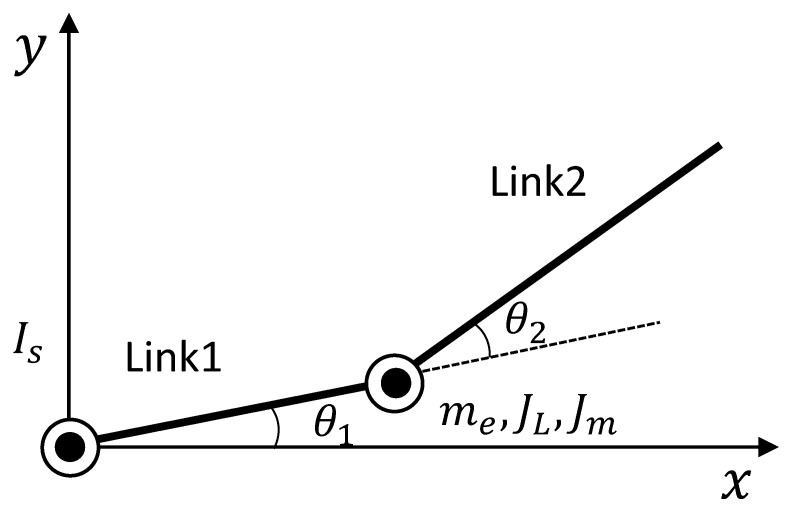
Robot arm used in this research (Conceptual diagram).

The dynamic model of the two-link robot arm that is the control target is shown in Figure 3. Lagrange’s equation of motion is used to derive the dynamic model. The equations of motion of Lagrangian and Lagrange are shown below.

(46) $$\begin{array}{c} L=K-V \end{array}$$

(47) $$\begin{array}{c} \frac{d}{dt}\left(\frac{\partial L}{\partial \dot{x}}\right)-\frac{\partial L}{\partial x}=F \end{array}$$

*K* is kinetic energy, *V* is potential energy, *x* is generalized coordinates and *F* is generalized force. The mechanical model derived from Lagrange’s equation of motion shown in (47) is expressed as the following equation [27]. (48) represents link 1 and (49) represents link 2.

(48) $$\begin{array}{cc} \; & m_{11}{\ddot{\theta }}_{1}+m_{12}{\ddot{\theta }}_{2}+\sum_{j=1}^{\infty }\left\{m_{14}\left(j\right){\ddot{v}}_{2j}\right\}+f_{1}+B_{1}{\dot{\theta }}_{1}=\tau_{1} \end{array}$$

(49) $$\begin{array}{cc} \; & m_{12}{\ddot{\theta }}_{1}+m_{22}{\ddot{\theta }}_{2}+\sum_{j=1}^{\infty }\left\{m_{24}\left(j\right){\ddot{v}}_{2j}\right\}+f_{2}+B_{2}{\dot{\theta }}_{2}=\tau_{2} \end{array}$$

The parameters used in (48) and (49) are shown in the following equations and Table 1.

(50) $$\begin{array}{cc} m_{11} & =I_{s}+I_{me}+I_{e}+m_{e}L_{1}^{2}+\int_{0}^{L_{1}}\rho_{1}A_{1}x_{1}^{2}dx_{1}+\int_{0}^{L_{2}}\rho_{2}A_{2}\left\{L_{1}^{2}+x_{2}^{2}+{\left(\sum_{j=1}^{\infty }\phi_{2j}\left(x_{2}\right)v_{2j}\right)}^{2}\right.\\ \; & \left.+2L_{1}x_{2}\cos\left(\theta_{2}\right)-2L_{1}\sum_{j=1}^{\infty }\phi_{2j}\left(L_{2}\right)v_{2j}\cos\left(\theta_{2}\right)\right\}dx_{2}\\ m_{12} & =I_{e}+\frac{\rho_{2}A_{2}L_{2}^{3}}{3}+\int_{0}^{L_{2}}\rho_{2}\left\{x_{2}^{2}\right.+{\left(\sum_{j=1}^{\infty }\phi_{2j}\left(x_{2}\right)v_{2j}\right)}^{2}\\ \; & +L_{1}x_{2}\cos\left(\theta_{2}\right)\left.-L_{1}\cos\left(\theta_{2}\right)\left(\sum_{j=1}^{\infty }\phi_{2j}\left(x_{2}\right)v_{2j}\right)\right\}dx_{2}\\ m_{14}\left(j\right) & =I_{e}+\int_{0}^{L_{2}}\rho_{2}A_{2}\left\{x_{2}\phi_{2j}\left(x_{2}\right)+L_{1}\phi_{2j}\left(x_{2}\right)\cos\left(\theta_{2}\right)\right\}dx_{2}\\ m_{22} & =\int_{0}^{L_{2}}\rho_{2}A_{2}\left\{x_{2}^{2}+{\left(\sum_{j=1}^{\infty }\phi_{2j}\left(x_{2}\right)v_{2j}\right)}^{2}\right\}dx_{2}\\ m_{24}\left(j\right) & =\int_{0}^{L_{2}}\rho_{2}A_{2}x_{2}\phi_{2j}\left(x_{2}\right)dx_{2}\\ f_{1} & =2\rho_{2}A_{2}\left({\dot{\theta }}_{1}+{\dot{\theta }}_{2}\right)\sum_{j=0}^{\infty }v_{2j}{\dot{v}}_{2j}+L_{1}\left(2{\dot{\theta }}_{1}+{\dot{\theta }}_{2}\right){\dot{\theta }}_{2}\cos\left(\theta_{2}\right)\sum_{j=0}^{\infty }\rho_{2}A_{2}\int_{0}^{L_{2}}\phi_{2j}\left(x_{2}\right)dx_{2}v_{2j}\\ \; & +2L_{1}\left({\dot{\theta }}_{1}+{\dot{\theta }}_{2}\right)\sin\left(\theta_{2}\right)\sum_{j=0}^{\infty }\rho_{2}A_{2}\int_{0}^{L_{2}}\phi_{2j}\left(x_{2}\right)dx_{2}{\dot{v}}_{2j}-\left(\frac{\rho_{2}A_{2}L_{1}L_{2}^{2}}{2}+m_{e}L_{1}L_{2}\right){\dot{\theta }}_{2}\sin\left(\theta_{2}\right)\\ f_{2} & =2\rho_{2}A_{2}\left({\dot{\theta }}_{1}+{\dot{\theta }}_{2}\right)\sum_{j=0}^{\infty }v_{2j}{\dot{v}}_{2j}+L_{1}{\dot{\theta }}_{1}^{2}\cos\left(\theta_{2}\right)\sum_{j=0}^{\infty }\rho_{2}A_{2}\int_{0}^{L_{2}}\phi_{2j}\left(x_{2}\right)dx_{2}v_{2j}\\ \; & +\left(\frac{\rho_{2}A_{2}L_{1}L_{2}^{2}}{2}+m_{e}L_{1}L_{2}^{2}+m_{e}L_{1}L_{2}\right){\dot{\theta }}_{1}^{2}\sin\left(\theta_{2}\right) \end{array}$$

#### 3.2.3. Hardware Configuration

**Figure 4:**
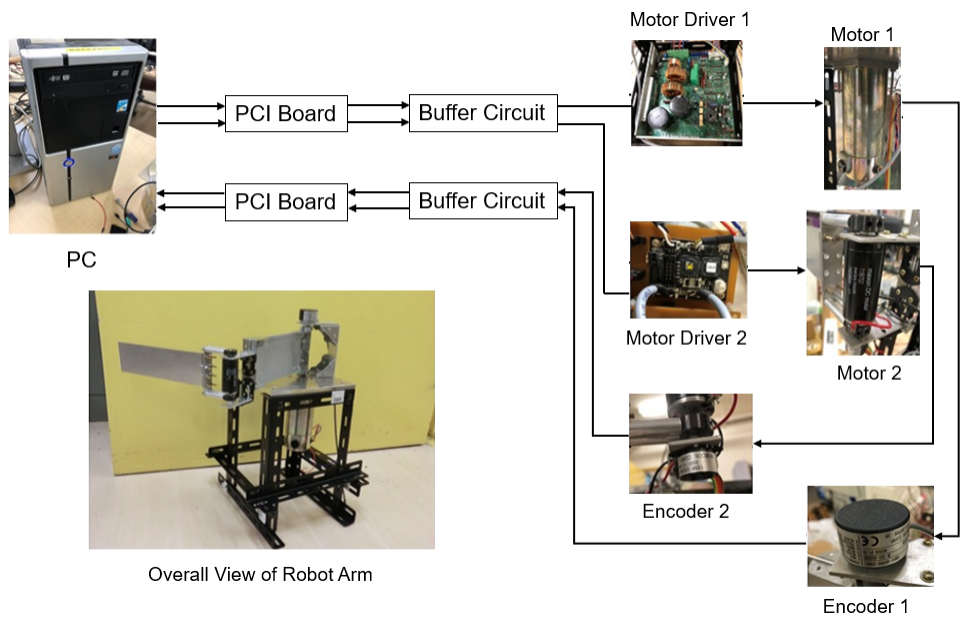
Proposed control system.

Figure 4 shows a schematic diagram of the experimental equipment. Motor 1 is TOYO TECHINICA DM-008D25F, motor 2 is maxon RE25 series 118752, encoder 1 is NEMICON 38H-4096-2MC and encoder 2 is NEMICON 18M-1024-2MC. The control program is written in C#. The command value calculated by the PC is DA-converted by PCI3521, then amplified twice by the buffer circuit and input to the servo amplifier. The voltage value is converted into a current value by the servo amplifier, and the current drives the motor to operate each link. The angle of each link is measured by capturing the number of pulses obtained from the encoder into a PC using the pulse counter board PCI6204.

#### 3.2.4. Problem Setup

Human arm multi-joint viscoelasticity is,

- Elucidation of the motor control principle of the brain that controls complex multi-joint movements,
- Quantitative understanding of motor deterioration caused by nerve and muscle disorders,
- It is considered to be an important factor in the development of human-friendly mechanical interfaces, and many studies have been conducted up to now.

Among them, there are many studies on the estimation of human arm multi-joint viscoelasticity and there are few examples of application to mechanical interfaces as in the third entry. The challenge in applying it to machine interfaces is the reproduction of voluntary movement components. The voluntary movement component is a feed-forward component output from a model based on experience in the brain when exercising.

Humans suppress disturbances with feedforward controls composed of the cerebellum. It is believed that the body is controlled by a feedback controller. The voluntary movement component represents the above feedforward component, and the human arm multi-joint viscoelasticity represents the feedback component. In the research to actually estimate the multi-joint viscoelasticity of the human arm, the estimation is performed after removing the above voluntary movement components with a filter. Therefore, when applying the multi-joint viscoelasticity of the human arm to the motion control of the robot arm, it is indispensable to reproduce the voluntary motion component. In the previous research, the motion control of the robot arm by the two-degree-of-freedom control system has been introduced using multi-joint viscoelasticity. A feedforward controller based on a mechanical model has the advantage that it is easy to design if the modeling of the system is completed, but it is easily affected by modeling errors. It may cause deterioration of control performance when conducting an actual machine experiment. In fact, in this study as well, when a control experiment using a feedforward controller based on a mechanical model was conducted, good control results can not be obtained due to things that are not taken into consideration during modeling, such as the influence of the dead zone of the motor driver. Therefore, in this research, we propose a feedforward controller based on SVR, which is one of machine learning methods. SVR is an application of SVM to a regression problem, and has features such as high generalization performance and effectiveness for non-linear input/output relationships. In addition, by learning the input/output relationships of the entire system including the motor driver as training data, it is possible to create a model that includes modeling errors and parts that were not considered during modeling. In this research, we design a feedforward controller using SVR and confirm its effectiveness in actual machine experiments.

## 4. Control System Design

**Figure 5:**
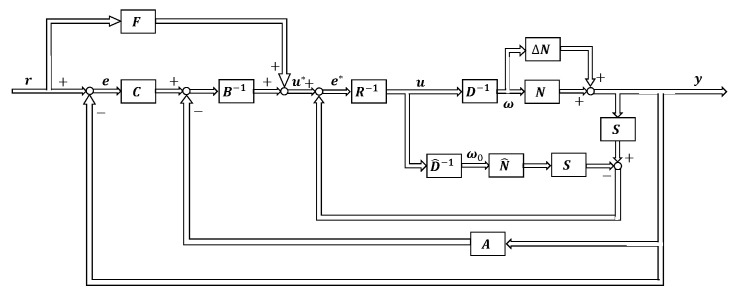
Block diagram of the proposed control system.

This section shows the proposed control system design method. Figure 5 shows the proposed control system. We design a two-degree-of-freedom control system by simulating the control system of the human body introduced in Section 3. The multi-joint viscoelasticity measured from humans is applied to the feedback controller *C*. The feedforward controller *F* based on SVR is also designed. In addition, in order to eliminate the influence and uncertainty due to interference inside the controlled object, the control system is designed based on the operator theory.

### 4.1. Controller Design Based on Multi-Joint Viscoelasticity

The follow-up controller *C* based on multi-joint viscoelasticity is shown by the following equation.

(51) C(e)=De˙+Re

R represents an elastic matrix and D represents a viscoelastic matrix. By applying this multi-joint viscoelastic matrix to a feedback controller, we aim to reproduce the same motion as humans. The elastic matrix and the viscosity matrix are 2-by-2 matrices and can be expressed as follows.

(52) $$\begin{array}{cc} \; & R=\left(\begin{array}{cc} R_{11} & R_{12}\\ R_{21} & R_{22} \end{array}\right)\\ \; & D=\left(\begin{array}{cc} D_{11} & D_{12}\\ D_{21} & D_{22} \end{array}\right) \end{array}$$

**Figure 6:**
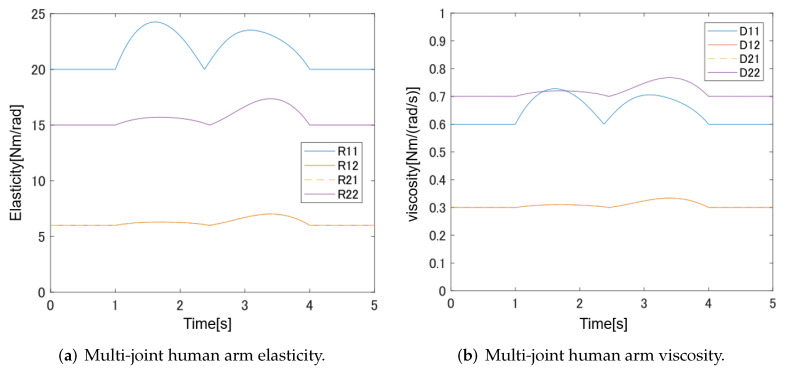
Multi-joint human arm elasticity and viscosity.

**Figure 7:**
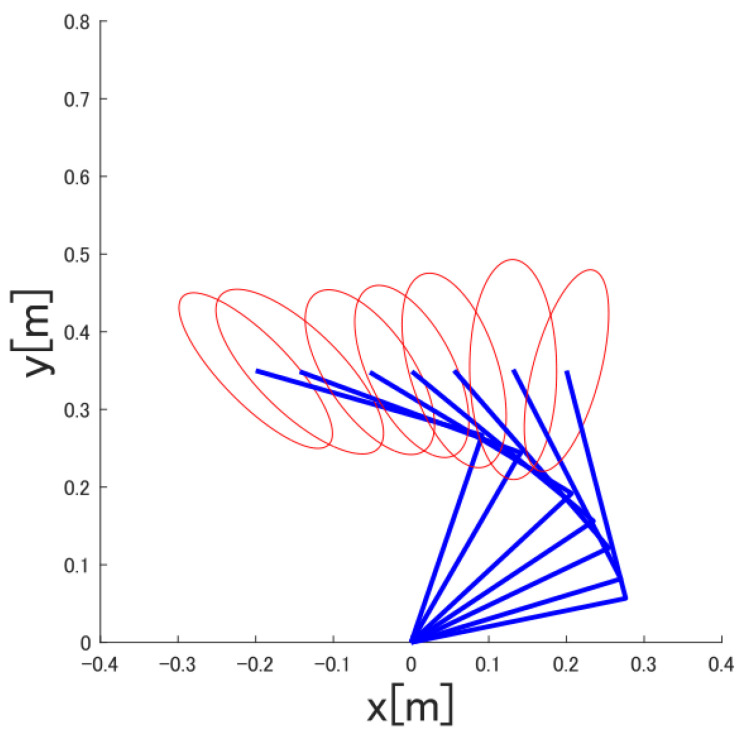
Multi-joint viscoelasticity.

The multi-joint human arm viscoelasticity used in this paper is shown in Figure 6. The combined movement of Link1 and Link2 produces translational movement, as shown in Figure 7.

### 4.2. Feedforward Controller Design Based on SVR

**Figure 8:**
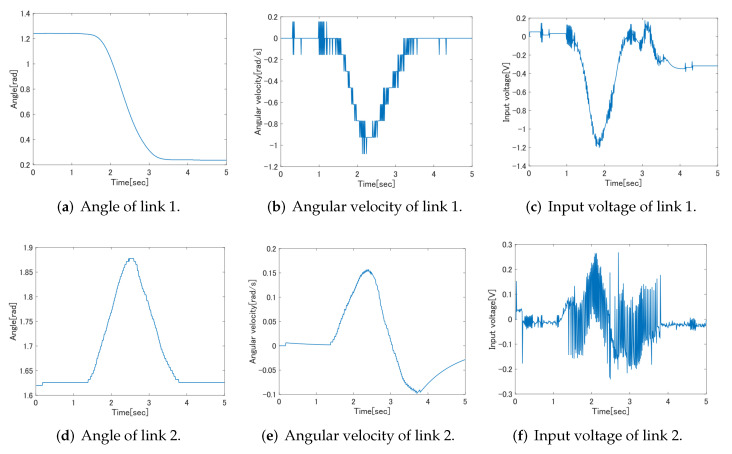
Training data of Link 1 and Link 2.

This section describes the design method of the feedforward controller based on SVR. Since SVR is one of the regression analysis methods, it is necessary to select training data. PD control is performed for the target trajectory used in the experiment in this study, and the input voltage applied to the motor driver of each link, the angle of each link and the angular velocity of each link at that time are measured as training data. The training data is shown in Figure 8.

SVR learning is performed based on the results shown in Figure 8. Since the angular velocity of link 2 was greatly affected by the measurement noise, the data filtered by the RC filter was trained as training data. In addition, the controller to be designed calculates an appropriate input voltage for the target angle and angular velocity. Therefore, the input/output relationship of the data to be learned by the SVR is the angle and angular velocity at the input and the voltage at the output. Note that it is the opposite of the normal input/output relationship.

### 4.3. Control System Design Based on Operator Theory

#### 4.3.1. Elimination of Uncertainty and Interference

The nominal model $\hat{P}$, which eliminates uncertainty and interference from other variables, can be expressed by the following equation.

(53) $$\hat{P}=\left\{\begin{array}{c} {\hat{m}}_{11}{\ddot{\theta }}_{1}+B_{1}{\dot{\theta }}_{1}=\tau_{1}\\ {\hat{m}}_{22}{\ddot{\theta }}_{2}+B_{2}{\dot{\theta }}_{2}=\tau_{2} \end{array}\right.$$

Using this nominal model, the operators *S* and *R* are designed to eliminate the effects and uncertainties caused by interference inside the controlled object [13]. By designing the controllers *S* and *R* as $S\hat{P}=I$, R=I, the uncertainty of the model and the influence of interference inside the controlled object can be eliminated, where *I* is an identity map. The following equation holds from the nonlinear feedback system shown in Figure 5.

(54) $$\begin{array}{cc} u^{*}\left(t\right) & -S\left(y\right)\left(t\right)+S\hat{N}{\hat{D}}^{-1}\left(u\right)\left(t\right)=R\left(u\right)\left(t\right)\\ u^{*}\left(t\right) & =S\left(y\right)\left(t\right)-S\hat{P}\left(u\right)\left(t\right)+R\left(u\right)\left(t\right)\\ \; & ={\hat{P}}^{-1}\left(y\right) \end{array}$$

**Figure 9:**
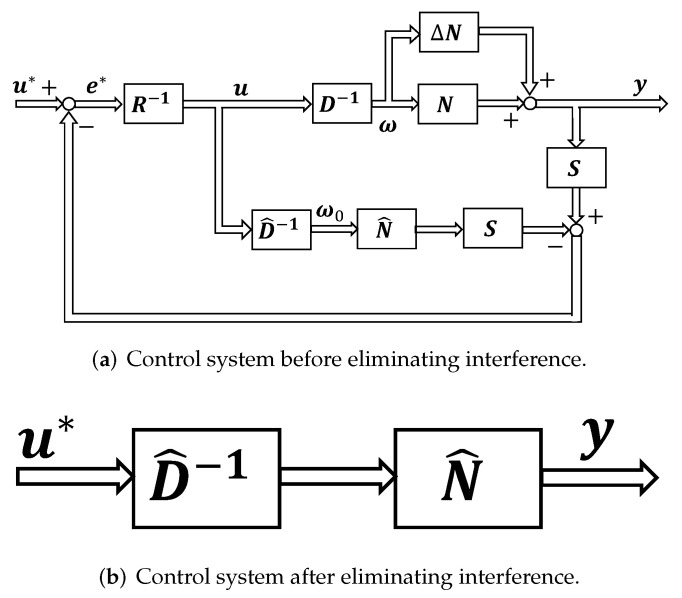
Control system before and after eliminating interference.

At this time, $y\left(t\right)=\hat{P}\left(u\right)\left(t\right)$, which shows that the uncertainty of the model and the influence of interference inside the controlled object can be eliminated. Equivalent feedback loops before and after removing uncertainty and interference are shown in Figure 9.

#### 4.3.2. Guarantee of Stability

In this section, the control system is designed based on operator theory, and the stability of the proposed control system is guaranteed. Specifically, based on operator theory, we design stabilization controllers *A* and $B^{-1}$ that satisfy the Bezout equation $A\hat{N}+B\hat{D}=I$. The right decomposition of the nominal plant $\hat{P}$ from which interference and uncertainty have been removed gives the following equation.

(55) $$\hat{N}=\left\{\begin{array}{c} {\hat{m}}_{11}{\ddot{y}}_{1}+B_{1}{\dot{y}}_{1}=\omega_{1}\\ {\hat{m}}_{22}{\ddot{y}}_{2}+B_{2}{\dot{y}}_{2}=\omega_{2}\\ \theta_{1}=y_{1},\theta_{2}=y_{2} \end{array}\right.$$

(56) $${\hat{D}}^{-1}=\left\{\begin{array}{c} \omega_{1}=\tau_{1}\\ \omega_{2}=\tau_{2} \end{array}\right.$$

The parameters used are shown below.

(57) $$\begin{array}{cc} {\hat{m}}_{11} & =I_{s}+I_{me}+I_{e}+m_{e}L_{1}^{2}+\int_{0}^{L_{1}}\rho_{1}A_{1}x_{1}^{2}dx_{1}\\ \; & +\int_{0}^{L_{2}}\rho_{2}A_{2}\left\{L_{1}^{2}+x_{2}^{2}+2L_{1}x_{2}\right\}dx_{2}\\ {\hat{m}}_{22} & =\int_{0}^{L_{2}}\rho_{2}A_{2}x_{2}^{2}dx_{2} \end{array}$$

Operator *A* is designed as follows:

(58) $$A\hat{N}=kI$$

From the Bezout equation, *B* can be expressed as follows.

(59) $$B=\left(1-k\right){\hat{D}}^{-1}$$

*k* is a design parameter. By constructing a nonlinear feedback system as shown in Figure 9b using operators *A* and *B*, the BIBO stability of the control system can be guaranteed.

## 5. Experiment

In this section, in order to confirm the effectiveness of the proposed control system, we verify it with an experimental device.

### 5.1. Experimental Conditions

Table 2 shows the parameters of the experimental equipment. The initial angle and target angle are the same as the angles used when the multi-joint viscoelasticity estimation was performed. Also, the sampling time was set to 0.01 s.

### 5.2. Selection of Hyperparameters of SVR

In this section, we select the hyperparameter *c* of SVR. There are three hyperparameters in SVR, and it is known that the regression model changes depending on the parameters. In this research, we focus on *c* among hyperparameters, experiment by changing the value of *c* step by step, and select the parameter with the best result. As the content of the experiment, for the proposed control system, only the hyperparameter *c* of the feedforward controller based on SVR is changed to 0.01, 0.05, 0.1, 1, 7, 10, and 100, and the experiment is performed. After that, the error between the experimental result and the target value is derived and evaluated by RMSE (root mean squared error). RMSE is expressed by the following equation.

(60) $$\mathrm{RMSE}=\sqrt{\frac{1}{n}\sum_{i=1}^{n}{\left(f_{i}-y_{i}\right)}^{2}}$$

**Figure 10:**
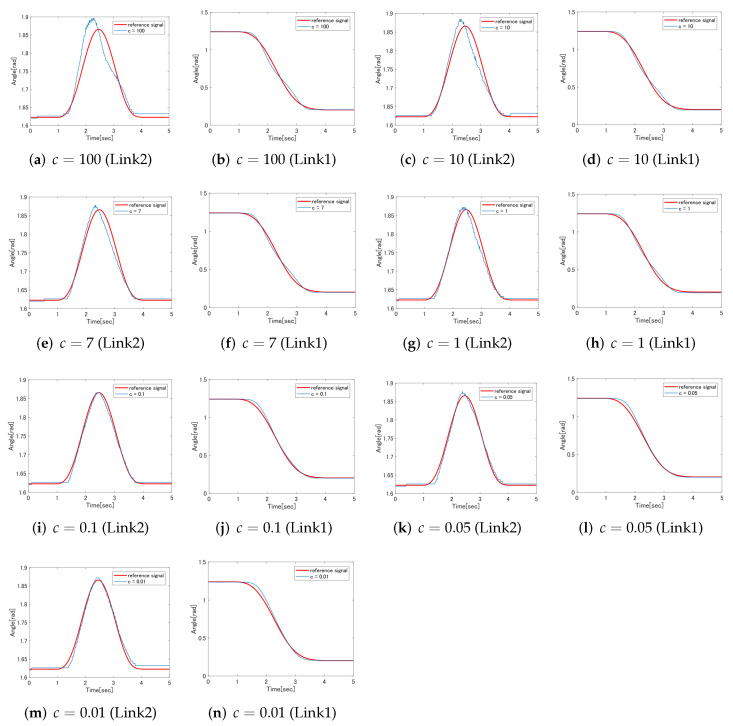
The experimental results.

Since the closer the RMSE is to 0, the smaller the error is, we select the hyperparameter *c* that minimizes the RMSE. The RMSE changes with different values of *c*, shown as Table 3. The experimental results are shown in Figure 10. Looking at the result of link 2, we can see that the control result changes depending on the value of hyperparameter *c*. When c=100, it can be seen from Figure 10 that a large overshoot occurs. If *c* is set to a large value, SVR is closer to the hard margin. While it is possible to create an accurate regression model that reflects most of the training data, it has the characteristic that noise during training data measurement is easily reflected in the regression model. Therefore, when a large value such as c=100 is set, a regression model that reflects the measurement noise contained in the training data is created, and it is considered that the overshoot shown in Figure 10a occurred. On the other hand, it can be confirmed that when *c* is made smaller, the above overshoot decreases and almost disappears at the time of c=0.1. It can be seen that when *c* is set to 0.1 or less, a slight delay occurs at the time of 3s to 4s due to the effect of the output from the SVR controller becoming smaller. Since the value of c=0.1 is also the minimum in the evaluation of RMSE, the *c* of link 2 is set to 0.1 in this study. For link 1, *c* is selected as 0.1 in the same way as link 2.

### 5.3. Experimental Results

In this section, we conduct experiments to confirm the effectiveness of the proposed control system and introduce the results. Specifically, in order to confirm the effectiveness of the proposed SVR-based feedforward controller, it is effective to compare the experimental results of the feedforward controller based on the dynamic model without the feedforward controller with the experimental results of the proposed method. In addition, the control systems other than the feedforward controller are the same, and the performance of the feedforward controller is compared. Figure 7 shows the elastic ellipsoid of the hand calculated from the multi-joint viscoelasticity used in the feedback controller. As in the previous section, RMSE is used for comparison of experimental results, and the best result is the experimental result with the smallest RMSE. Table 4 shows the RMSE results.

**Figure 11:**
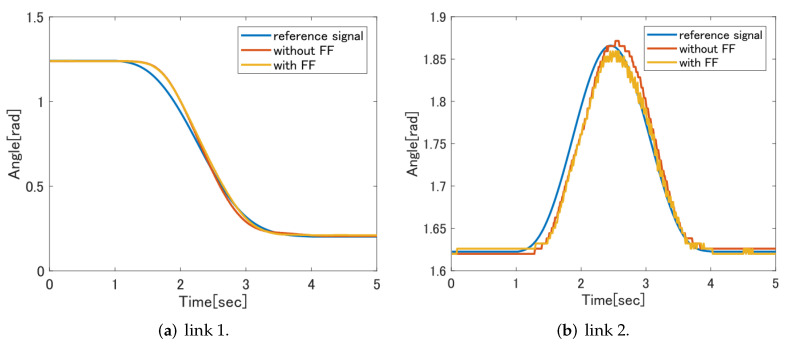
Comparison of FF without controller and dynamic model with FF controller.

Figure 11 shows a comparison of the control results between the feedforward controller without the feedforward controller and the feedforward controller based on the dynamic model. In the result of link 2, it can be confirmed that the tracking performance is improved from 2.5 s to 3 s. The result of RMSE is also smaller in the feedforward controller based on the dynamic model than in the case without the feedforward controller, and the effectiveness of the feedforward controller can be confirmed. On the other hand, for Link 1, no significant improvement is seen in the control results, and the analysis results by RMSE did not change much. The cause is thought to be the error that occurred during modeling.

**Figure 12:**
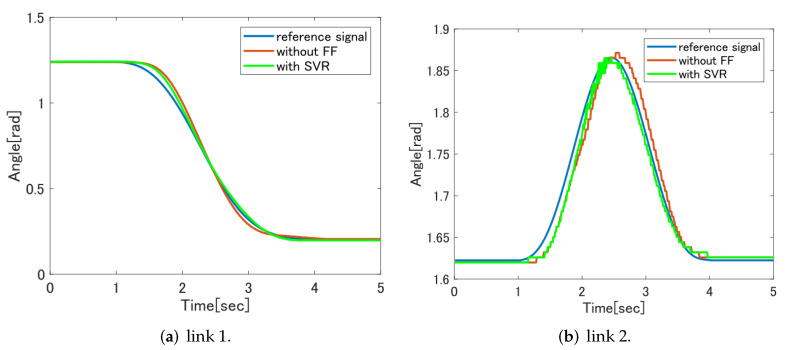
Comparison of FF without controller and SVR FF with controller.

Figure 12 shows a comparison of the control results between the feedforward controller without the feedforward controller and the feedforward controller based on SVR. It can be confirmed that the tracking performance is improved for both link 1 and link 2. The analysis result by RMSE is also smaller in the feedforward controller based on SVR than in the case without the feedforward controller, and the effectiveness of the feedforward controller can be confirmed.

**Figure 13:**
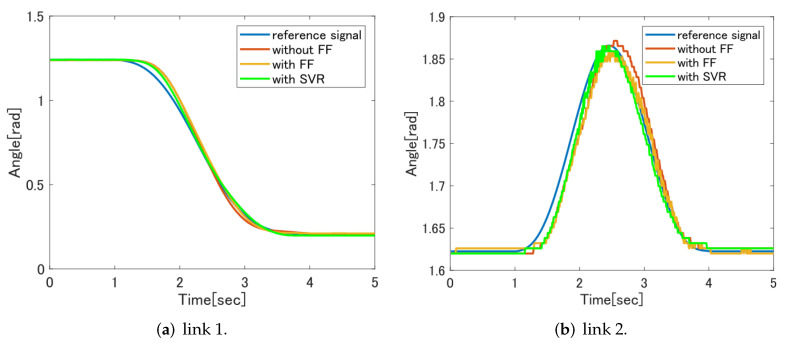
Comparison of all control results.

Figure 13 shows a comparison of the control results of the feedforward controller without the feedforward controller, the feedforward controller based on the dynamic model and the feedforward controller based on the SVR. Comparing the control results of the feedforward controller based on the dynamic model and the feedforward controller based on SVR, it is confirmed that the feedforward controller based on SVR follows the target value more for both link 1 and link 2. The RMSE value is also lower in the feedforward controller based on SVR, and it can be seen that the feedforward controller based on SVR has better tracking performance numerically. It is considered that this is because it was possible to create a model closer to the experimental equipment by creating a model from the training data of the actual machine experiment using SVR.

**Figure 14:**
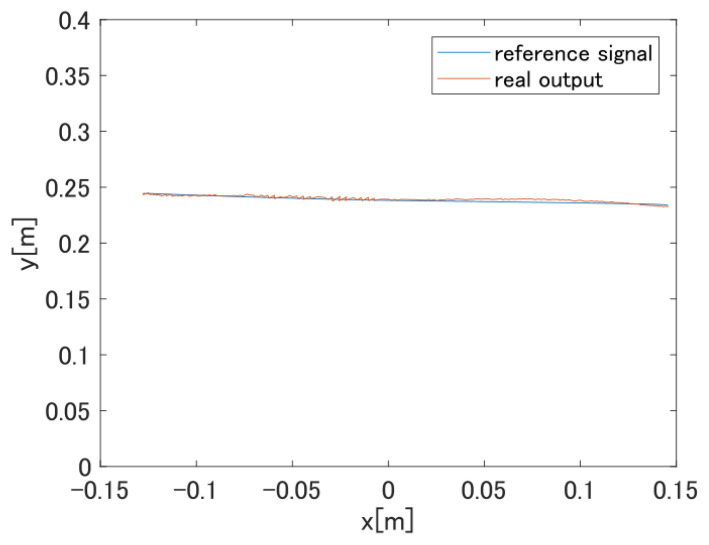
Multi-joint arm movement trajectory.

Figure 14 shows the target value of the hand position coordinates and the actual output. With the proposed control system, we are able to confirm the follow-up of the hand. From the above, we are able to verify the effectiveness of the feedforward controller based on the proposed SVR method in actual machine experiments.

## 6. Conclusions

In this study, we propose a two-degree-of-freedom control system using multi-joint viscoelasticity, and conduct a motion control experiment of a two-link robot arm. Focusing on the feedforward controller in the two-degree-of-freedom control system, we propose a feedforward controller based on SVR, which is one of machine learning methods. Finally, the effectiveness of the proposed method is verified by an actual machine experiment. The controller is designed as a multi-joint arm like one and it is based on the characteristic of the human arm multi-joint viscoelasticity. The characteristic is analyzed from the experiment.

In the future, some intelligent control as well as adaptive control methods [28,29,30] can be considered to further improve the current work.

## Figures and Tables

**Table 1:** Parameters of each link.

$\rho_{1}$	Link1 density	[kg/m${}^{3}$]
$\rho_{2}$	Link2 density	[kg/m${}^{3}$]
$L_{1}$	Link1 length	[m]
$L_{1}$	Link2 length	[m]
$A_{1}$	Link1 cross-sectional area	[m${}^{2}$]
$A_{2}$	Link2 cross-sectional area	[m${}^{2}$]
$I_{s}$	Moment of inertia of rotor	[kg/m${}^{2}$]
$J_{L}$	Moment of inertia of set collar	[kg/m${}^{2}$]
$J_{m}$	Moment of inertia of rotor	[kg/m${}^{2}$]
$\tau_{1}$	Torque applied to Link1	[N·m]
$\tau_{2}$	Torque applied to Link1	[N·m]

**Table 2:** Laboratory equipment parameters.

$\rho_{1}$	Link1 density	8030 kg/m${}^{3}$
$\rho_{2}$	Link2 density	8030 kg/m${}^{3}$
$L_{1}$	Link1 length	0.2 m
$L_{1}$	Link2 length	0.2 m
$A_{1}$	Link1 cross-sectional area	127.5 mm${}^{2}$
$A_{2}$	Link2 cross-sectional area	25 mm${}^{2}$
$I_{s}$	Moment of inertia of rotor	7.33 × 10${}^{-6}$ kg·m${}^{2}$
$J_{L}$	Moment of inertia of set collar	8.71 × 10${}^{-9}$ kg·m${}^{2}$
$J_{m}$	Moment of inertia of rotor	1.08 × 10${}^{-6}$ kg·m${}^{2}$
$E_{2}I_{2}$	Flexural rigidity of the arm	359 N·m${}^{2}$
$C_{1}$	Attenuation coefficient	1.88 × 10${}^{-5}$
*B*	Viscous friction coefficient	2.1 × 10${}^{-3}$ N·s/m
$K_{t1}$	Torque constant of motor 1	0.38 N·m/A
$K_{t2}$	Torque constant of motor 2	0.0234 N·m/A

**Table 3:** RMSE results with different value of *c*.

The Value of *c*	RMSE (Link 1)	RMSE (Link 2)
100	0.0263	0.0263
10	0.0214	0.0166
7	0.206	0.0131
1	0.0200	0.0108
0.1	0.0188	0.0065
0.05	0.0226	0.0069
0.01	0.0250	0.0085

**Table 4:** RMSE results.

FF Controller	RMSE (Link 1)	RMSE (Link 2)
None	0.0271	0.0142
Mechanical model	0.0273	0.0136
SVR	0.206	0.0174

## Data Availability

Data are not publicly available due to privacy considerations.

## References

[1] Hogan N. (1985). Impedance Control: An approach to manipulation. J. Dyn. Syst. Meas. Control.

[2] Kearney R.E., Hunter I.W. (1982). Dynamics of human ankle stiffness: Variation with displacement amplitude. J. Biomech..

[3] Gomi H., Kawato M. (1996). Equilibrium-point control hypothesis examined by mesured arm-stiffness during multijoint movement. Science.

[4] Deng M., Teramura Y., Wang A., Yanou A. Particle filter-based viscoelasticity estimation of human multi-joint arm. Proceedings of the 18th World Congress of IFAC.

[5] Deng M., Inoue A., Zhu Q. (2011). An integrated study procedure on real time estimation of time varying multijoint human arm viscoelasticity. Trans. Inst. Meas. Control.

[6] Deng M., Saijo N., Gomi H., Inoue A. (2006). A robust real time method for estimating human multijoint arm viscoelasticity. Int. J. Innov. Comput. Inf. Control.

[7] Deng M., Inoue A., Gomi H., Hirashima Y. (2006). Recursive filter design for estimating time varying multijoint arm viscoelasticity. Int. J. Comput. Syst. Signal.

[8] Deng M., Bu N., Yanou A. Framework of an estimation algorithm of time varying multijoint human arm viscoelasticity. Proceedings of the 3rd International Conference on Bio-inpsired Systems and Signal Processing.

[9] Gomi H., Kawato M. (1997). Human arm stiffness and equilibrium-point trajectory during multi-joint movement. Biol. Cybern..

[10] Kataguchi T., Deng M., Noge Y. Online Human Multi-joint Arm’s Viscoelasticity Estimation during Movement. Proceedings of the 2019 International Conference on Advanced Mechatronic Systems.

[11] Wang A., Deng M. (2012). Operator-based robust nonlinear tracking control for a human multi-joint arm-like manipulator with unknown time-varying delays. Appl. Math. Inf. Sci..

[12] Gomi H., Konno T. Real time estimation of time-varaying human multijoint arm viscoelasticity during movement. Proceedings of the 20th Annual International Conference of the IEEE Engineering in Medicine and Biology Society.

[13] Wang A., Deng M. (2013). Robust nonlinear multivariable tracking control design to a manipulator with unknown uncertainties using operator-based robust right coprime factorization. Trans. Inst. Meas. Control.

[14] Vladimir N.V. (1998). Statistical Learning Theory.

[15] Jiang L., Deng M., Inoue A. (2009). Obstacle Avoidance and Motion Control of a Two Wheeled Mobile Robot Using SVR Technique. Int. J. Innov. Comput. Inf. Control.

[16] Cristianini N., Shawe-Taylor J. (2000). An Introduction to Support Vector Machines and Other Kernel-Based Learning Methods.

[17] Cherkassky V., Mulier F. (1998). Learning from Data: Concepts, Theory, and Methods.

[18] LIBSVM—A Library for Support Vector Machines. http://www.csie.ntu.edu.tw/cjlin/libsvm/.

[19] Deng M. (2014). Operator-Based Nonliner Control Systems Design and Applications.

[20] Deng M., Inoue A., Ishikawa K. (2006). Operator-based nonlinear feedback control design using robust right coprime factorization. IEEE Trans. Autom. Control.

[21] Furukawa K., Deng M. Operator based Fault Detection and Compensation Design of an Unknown Multivariable Tank Process. Proceedings of the 2013 International Conference on Advanced Mechatronic Systems.

[22] Umemoto K., Matsuno F., Deng M. Control system design for nonlinear uncertain plants using robust right coprime factorization. Proceedings of the 12th Society of Instrument and Control Engineers, Control Department Competition.

[23] Chen G., Han Z. (1998). Robust right coprime factorization and robust stabilization of nonlinear feedback control systems. IEEE Trans. Autom. Control.

[24] Bi S., Deng M., Xiao Y. (2015). Robust Stability and Tracking for Operator-based Nonlinear Uncertain Systems. IEEE Trans. Autom. Sci. Eng..

[25] Feldman A. (1966). Functional tuning of nervous system with control of movement or maintenance of a steady posture, II. Controllable parameters of the muscles. Biophysics.

[26] Feldman A.G. (1966). Functional tuning of nervous system with control of movement or maintenance of a steady posture, II. Mechanographic analysis of execution by man of the simplest motor task. Biophysics.

[27] Cheol H., Choi S. (2001). Position control of a two-link flexible manipulator featuring piezoelectric and sensors. Mechatronics.

[28] Tavoosi J., Shirkhani M., Abdali A., Mohammadzadeh A., Nazari M., Mobayen S., Bartoszewicz A. (2021). A New General Type-2 Fuzzy Predictive Scheme for PID Tuning. Appl. Sci..

[29] Tavoosi J. (2021). Intelligent Model Predictive Control for Boiler Temperature. Autom. Control Comput. Sci..

[30] Mo H., Farid G. (2019). Nonlinear and adaptive intelligent control techniques for quadrotor uav—A survey. Asian. J. Control.

